# Editorial: Cerebrovascular imaging—From micro- to macroscopic scales

**DOI:** 10.3389/fnins.2022.1086022

**Published:** 2022-11-22

**Authors:** Chengcheng Zhu, Mickael Tanter, Zhaoyang Fan, Song Hu, Umar Sadat, Danny J. J. Wang

**Affiliations:** ^1^Department of Radiology, University of Washington, Seattle, WA, United States; ^2^INSERM U1273 Physics for Medicine Paris (ESPCI), Paris, France; ^3^Department of Radiology, University of Southern California, Los Angeles, CA, United States; ^4^McKelvey School of Engineering, Washington University in St. Louis, St. Louis, MO, United States; ^5^Department of Surgery, University of Cambridge, Cambridge, United Kingdom; ^6^Department of Neurology, University of Southern California, Los Angeles, CA, United States

**Keywords:** stroke, vessel wall imaging, MRI, perfusion, atherosclerosis, aneurysm

Neurovascular diseases, including stroke, atherosclerosis, aneurysm, cerebral small vessel disease (cSVD), vascular cognitive impairment and dementia (VCID), and others, are major causes of death and disabilities. The prevalence of these neurovascular diseases has been steadily increasing due to the global population aging. During the past few decades, the technical advances of medical imaging methods, including MRI, CT, PET, optical and ultrasound (US) imaging, as well as photoacoustic imaging, have given the opportunities to image the neurovascular system in great details from micro to macro scales, from the capillaries (<10 um), arterioles/venules (~100 um) to large vessels (mm) including lumen, vessel wall and perivascular space.

## Vessel wall imaging

While most of the vascular imaging methods focus on the flowing lumen, vessel wall MRI provides the ability to directly visualize the vessel wall, which is the source of pathology in atherosclerotic plaques, aneurysms and other vasculopathy. Most vessel MRI studies were performed at 3 Tesla due to its high signal to noise ratio (SNR) compared to 1.5 Tesla, and the use of ultra-high field 7 Tesla MRI scanners further increase the ability (Zhu et al., 2016; Rutland et al., 2020).

In this Research Topic collection, Zhang L. et al. developed a T2 prepared inversion recovery (IR) MRI sequence (T2IR-SPACE) which markedly suppressed the Cerebrospinal Fluid (CSF) signal without much SNR loss of the other tissues (i.e., vessel wall, white matter, and gray matter). Such sequence can be used in multi-contrast intracranial vessel wall imaging to achieve good CSF suppression and improve the vessel wall contrast. Kong et al. developed 3D inner-volume (IV) TSE (SPACE) sequence with optimized 2D spatially selective excitation (SSE) radio frequency (RF) pulses, and they achieved the highest resolution (0.3 mm isotropic) *in vivo* vessel wall MRI so far by using a 7T scanner. They found clearer delineation of lenticulostriate artery (LSA) than conventional SPACE images.

Xu et al. developed automatic segmentation methods for carotid and intracranial plaques on vessel wall MRI in 124 patients using machine learning. Their method demonstrated satisfactory agreement with the manual method, with dice values of 93.8% for lumen contours and 86.0% for outer wall contours, which were higher than those obtained from the traditional U-Net, Attention U-Net, and Inception U-Net. Lindenholz et al. found an interrelationship between large vessel wall lesion burden and cerebral parenchymal manifestations often linked to small vessel disease (SVD). Li J. et al. studied 68 patients with vertebrobasilar atherosclerosis using vessel wall MRI, and found the vertebrobasilar junction (VBJ) angle over 90° might aggravate the vessel wall condition of the atherosclerotic vertebrobasilar arteries, which might serve as a potential risk factor for vertebrobasilar atherosclerosis.

Eisenmenger et al. presented 9 cases of arteriovenous malformation (AVM) and found Vessel wall “enhancement” occurs in AVMs with no prior clinical rupture. Xiao et al. presented a case of intracranial plaque with serial vessel wall MRI follow up and they found vessel wall MRI can directly visualize the morphology and signal change of plaques. The suggest early identification of patients who do not respond well to medication is critical to prevent the recurrence of cardiovascular events in these patients.

## Perfusion

Perfusion imaging plays an important role in the management of patients with neurovascular diseases. Jann et al. applied 3D pseudo-continuous arterial spin labeling (pCASL) MRI in the cohort of elderly Latinx subjects and found cerebral blood flow (CBF) in the leptomeningeal and perforator middle cerebral artery (MCA) territories were the most likely candidate biomarker of Vascular Cognitive Impairment and Dementia (VCID). Shi et al. compared several software for CT perfusion measurements and found F-STROKE software had excellent agreement with the widely used analysis tool of RAPID in measuring ischemic core volume (ICV) and penumbra volume (PV). Shou et al. developed super-resolution perfusion imaging and achieved high spatial resolution (isotropic-2 mm) using 2D simultaneous multi-slice (SMS) pseudo-continuous arterial spin labeling (pCASL) and slice dithered enhanced resolution (SLIDER) technique. de Bortoli et al. used T2^*^-weighted imaging and ultra-small superparamagnetic iron oxide nanoparticles to obtain subtraction angiographies and steady-state cerebral blood volume (ss-CBV) maps. They found the maps agreed well with first pass dynamic susceptibility contrast MRI (DSC-MRI). They concluded that iron oxide nanoparticle-based ss-CBV could serve as a robust, non-invasive imaging surrogate marker for neocortical vessels, with the potential to reduce and refine preclinical models targeting the development and outgrowth of cerebral collateralization.

## Hemodynamics

Hemodynamics plays important roles in the development and progression of vascular diseases. The hemodynamics information could be either computed using computational fluid dynamics (CFD) by using image-derived geometry or directly acquired by flow imaging. Zhai et al. performed CFD analysis in 20 unruptured and 12 ruptured pericallosal artery aneurysms (PAA) with 3D digital subtraction angiography (DSA), and found a high mean oscillatory shear index (OSI) was an independent risk factor for PAA rupture. Zhang G. et al. studied the Hemodynamics using 4D flow MRI and found increased WSS, especially during the diastolic period and in the axial direction, may be a signal of a high-risk plaque and may cause cerebrovascular events in patients with moderate carotid artery stenosis.

## Deeping learning in neurovascular imaging

Zhu et al. studied a total of 632 patients with 668 MCA aneurysms (423 ruptured aneurysms) from five hospitals and quantified their radiomics and morphological features from computed tomography angiography images. They concluded integrating radiomics features into conventional models might provide additional value in ruptured MCA aneurysms classification. Shou et al. applied deep learning methods in perfusion imaging, and Xu et al. applied deep learning in vessel wall imaging.

## Other research

Bretzner et al. analyzed a multi-site cohort of 4,163 acute ischemic strokes (AIS) patients with T2-FLAIR MR images with total brain and white matter hyperintensity (WMH) segmentations. They found Radiomics extracted from T2-FLAIR images of AIS patients capture microstructural damage of the cerebral parenchyma and correlate with clinical phenotypes, suggesting different radiographical textural abnormalities per cardiovascular risk profile. Li T. et al. found high-resolution flat-detector computed tomography (HR-FDCT) improves visualization of the fine structures of intracranial stents deployed for symptomatic intracranial atherosclerotic stenosis (ICAS) compared with that visualized using conventional flat-detector computed tomography (FDCT). The concluded HR-FDCT improves assessment of stent deployment and could reduce the risk of complications. Du et al. reviewed the histological and imaging features of intimal and medial calcification within the large intracranial arteries and highlighted its clinical relevance.

## Future direction

Vessel wall imaging is a hot topic with increasing research and clinical translation interests (Mossa-Basha et al., 2022) in the past decade and we include seven articles in this Research Topic. However, most of current studies were cross-sectional with limited sample sizes. Future larger scale longitudinal studies and randomized controlled trails are needed to establish vessel wall imaging markers to predict patients' outcome (Zhu and Mossa-Basha, 2021). Perfusion imaging with noninvasive ASL techniques continues to grow and translate into clinical use. Hemodynamic conditions in neurovascular disease have been studied for several decades but the clinical utility still need larger scale study to prove. Recent advances in 4D flow imaging with higher resolution and shorter scan time may facilitate its clinical translation. Deep learning is another hot topic in neurovascular imaging recently and it has great potential for image acceleration, reconstruction and automating image analysis.

## Author contributions

All authors listed have made a substantial, direct, and intellectual contribution to the work and approved it for publication.

## Funding

CZ was supported by US National Institute of Health (NIH) grants R01HL162743 and R00HL136883. ZF was supported by NIH grant R01HL147355.

## Conflict of interest

The authors declare that the research was conducted in the absence of any commercial or financial relationships that could be construed as a potential conflict of interest.

## Publisher's note

All claims expressed in this article are solely those of the authors and do not necessarily represent those of their affiliated organizations, or those of the publisher, the editors and the reviewers. Any product that may be evaluated in this article, or claim that may be made by its manufacturer, is not guaranteed or endorsed by the publisher.
